# Targeting of non-oncogene addiction

**DOI:** 10.18632/aging.100796

**Published:** 2015-08-18

**Authors:** Ana Igea, Jalaj Gupta, Angel R. Nebreda

**Affiliations:** Institute for Research in Biomedicine (IRB Barcelona), 08028 Barcelona, Spain

Signaling pathways control all phases of tumor development and are critical in cancer therapy as they are largely responsible for the ability of tumor cells to survive or die in response to chemotherapy and radiotherapy. The p38 MAPK signaling pathway is one of the routes that cells use extensively to interpret extracellular signals and orchestrate appropriate responses. This pathway was originally characterized as a key regulator of stress and inflammatory processes, which prompted the development of chemical inhibitors mainly targeting the p38α and p38β family members. These inhibitors were expected to curtail production of inflammatory mediators and be useful for the treatment of inflammatory diseases such as rheumatoid arthritis. Unfortunately, the available information indicates rather disappointing outcomes, sometimes due to toxicity and in other cases for lack of efficacy, notwithstanding that some clinical trial results are not made public [1]. However, recent clinical trials with p38 MAPK inhibitors have given promising results for Chronic Obstructive Pulmonary Disease [2].

Intensive research over the past two decades has provided good evidence for the implication of the p38 MAPK pathway in cellular processes unrelated to stress that are important for normal physiology. It is now clear, for example, that p38 MAPK can regulate the proliferation, differentiation and survival of many cell types. In addition, p38 MAPK signaling has been implicated in several pathologies including cancer. Initial studies performed using cell lines both in culture and in mouse xenografts indicated that this pathway can suppress tumorigenesis. More recent studies have included the use of genetically modified mice to address the role of p38 MAPK signaling in different cell types, showing that this pathway can regulate tumor development at different levels.

Our group has contributed to the study of how the p38 MAPK pathway regulates tumor initiation and progression *in vivo*. Using mouse models, we have shown an important role of p38 MAPK signaling in colon and breast cancer [3, 4]. As reported for other tumor types, we provided evidence that the p38 MAK pathway suppresses tumor initiation in a mouse model of inflammation-associated colon tumorigenesis. Unexpectedly, once the tumor is formed, p38 MAPK signaling contributes to the proliferation and survival of the malignant cells and inhibition of p38 MAPK reduces colon tumor growth [3]. Moreover, genetic and pharmacological experiments indicate that p38 MAPK inhibition cooperates with chemotherapeutic drugs such as cisplatin to kill breast and colon cancer cells in culture and to reduce tumor size *in vivo* in a mouse model of breast cancer [4]. Along the same lines, other groups have shown that inhibition of the p38 MAPK pathway potentiates the anti-tumoral effects of doxorubicin and sorafenib in mouse models of lung and liver cancer, respectively [5, 6]. Taken together, these results strongly suggest that p38 MAPK inhibitors can be potentially exploited for cancer therapy in combination with chemotherapeutic drugs.

The results obtained in mouse models of cancer are promising but any attempt to modulate p38 MAPK activity for therapeutic purposes should be carefully evaluated in preclinical models. This is always an important validation step but in the case of p38 MAK signaling is critical, given the many functions that this pathway can perform depending on the cellular context. We have started to use patient-derived xenografts (PDX) as preclinical models that recapitulate the complexity and heterogeneity of the human tumors. Using PDX models, we have confirmed that pharmacological inhibition of p38 MAPK impairs the growth of colon tumors derived from patients [7]. In line with the possible therapeutic interest that inhibition of p38 MAPK signaling could have for colon cancer treatment, p38 MAPK inhibitors either alone or in combination with other drugs have been used or are currently in clinical trials for different types of cancer (https://clinicaltrials.gov).

It therefore seems that tumor cells may become addicted to p38 MAPK signaling, perhaps to be able to tolerate homeostatic control deficiencies and the kind of permanent stressful conditions in which they have to thrive. Considering that sustained activation of the p38 MAPK pathway in normal cells usually leads to cell cycle arrest and apoptosis, it cannot be considered an oncogenic route. However, the ability of this pathway to perform a variety of functions makes tumor cells to rely on it, illustrating a good example of non-oncogene addiction.

In summary, results obtained by our group and others support that the p38 MAPK pathway could act as an accessory component of oncogenic networks, which can be potentially exploited in combination therapies to effectively shut down pro-tumorigenic pathways and facilitate tumor cell death. Thus, pharmacological inhibitors of p38 MAPK are worth exploring for cancer therapy and combined with chemotherapeutic drugs could improve current treatments and reduce side effects.

## References

[1] Coulthard LR (2009). Trends Mol Med.

[2] Norman P (2015). Expert Opin Investig Drugs.

[3] Gupta J (2014). Cancer Cell.

[4] Pereira L (2013). EMBO Mol Med.

[5] Morandell S (2013). Cell Rep.

[6] Rudalska R (2014). Nature Med.

[7] Gupta J (2015). Oncotarget.

